# Assessing the reliability of eBURST using simulated populations with known ancestry

**DOI:** 10.1186/1471-2180-7-30

**Published:** 2007-04-12

**Authors:** Katherine ME Turner, William P Hanage, Christophe Fraser, Thomas R Connor, Brian G Spratt

**Affiliations:** 1 Department of Infectious Disease Epidemiology, Imperial College, St. Mary's Hospital Campus, Norfolk Place, London W2 1PG, UK

## Abstract

**Background:**

The program eBURST uses multilocus sequence typing data to divide bacterial populations into groups of closely related strains (clonal complexes), predicts the founding genotype of each group, and displays the patterns of recent evolutionary descent of all other strains in the group from the founder. The reliability of eBURST was evaluated using populations simulated with different levels of recombination in which the ancestry of all strains was known.

**Results:**

For strictly clonal simulations, where all allelic change is due to point mutation, the groups of related strains identified by eBURST were very similar to those expected from the true ancestry and most of the true ancestor-descendant relationships (90–98%) were identified by eBURST. Populations simulated with low or moderate levels of recombination showed similarly high performance but the reliability of eBURST declined with increasing recombination to mutation ratio. Populations simulated under a high recombination to mutation ratio were dominated by a single large straggly eBURST group, which resulted from the incorrect linking of unrelated groups of strains into the same eBURST group. The reliability of the ancestor-descendant links in eBURST diagrams was related to the proportion of strains in the largest eBURST group, which provides a useful guide to when eBURST is likely to be unreliable.

**Conclusion:**

Examination of eBURST groups within populations of a range of bacterial species showed that most were within the range in which eBURST is reliable, and only a small number (e.g. *Burkholderia pseudomallei *and *Enterococcus faecium*) appeared to have such high rates of recombination that eBURST is likely to be unreliable. The study also demonstrates how three simple tests in eBURST v3 can be used to detect unreliable eBURST performance and recognise populations in which there appears to be a high rate of recombination relative to mutation.

## Background

In recent years there has been increasing emphasis on the use of digital data to characterise strains of bacterial species. Multiple single nucleotide polymorphisms and multiple variable number tandem repeats have been used for digital strain characterisation of species that genetically are highly uniform [1-5] and multilocus sequence typing (MLST) has been used widely for more variable species [6,7]. In MLST, the relatedness among strains is typically displayed as a dendrogram, based on differences in allelic profiles, which identifies clusters of similar strains but provides no information on ancestry and patterns of descent among the strains within the clusters. The sequences of the MLST loci can also be used to explore relationships among strains but recombination occurs frequently in many bacterial species and impacts on the ability of sequence data to discern the true relationships among strains [8]. Consequently, new methods to explore recent evolutionary history that are less subject to distortions introduced by recombination have been developed.

One popular method, eBURST, was designed for the analysis of MLST data, although it can be used with other types of digital data [9,10]. eBURST incorporates a simple model of bacterial evolution in which strains increasing in frequency (under selection or drift) diversify to form clusters of similar genotypes descended from the founding strain. In terms of MLST, isolates of an expanding founding strain (founding sequence type; ST) initially have the same allelic profile, but diversification results in the appearance of variants in which one of the MLST loci has changed (single locus variants; SLVs), either as the result of mutation or recombination. Further diversification generates double locus variants (DLVs) and then triple locus variants (TLVs) of the founding ST, to result in a cluster of closely related STs descended from the founding ST (a clonal complex).

In eBURST a clonal complex is defined as a group of STs in a population that share 6/7 alleles with at least one other ST in the group. The BURST algorithm identifies these clonal complexes within bacterial populations (eBURST groups), infers the founding ST of each clonal complex, and displays the likely pattern of recent evolutionary descent of all STs within the clonal complex from this predicted founder [11]. Founder STs are assigned as the ST in an eBURST group that is linked to the greatest number of SLVs, with confidence in this assignment evaluated by bootstrapping [11], and lines drawn between SLVs (links) in an eBURST diagram identify inferred ancestor-descendant relationships. Consequently, eBURST groups are typically radial, with the founder ST linked to all its SLVs, which may themselves be linked to DLVs of the founder and so on. The nature of the allelic change (mutation versus recombination) is unimportant for discerning patterns of descent among related STs within a clonal complex and therefore, for exploring recent ancestry, eBURST is uninfluenced by recombination, in contrast to most methods that use the nucleotide sequences themselves [11].

The eBURST program (freely available online [12]) is widely used but the reliability of the method for identifying groups of strains descended from a recent common ancestor, and patterns of recent descent, has not been evaluated. Bacterial populations can vary greatly in their level of genetic diversity and in the extent to which allelic change is mediated by recombination compared to point mutation [13]. Intuitively, eBURST would be expected to perform well in cases where all allelic changes occur by point mutation (the strictly clonal situation), but its performance with populations in which an increasing proportion of allelic change occurs by recombination needs to be assessed. Evaluating the performance of eBURST is not possible using empirical data since the true evolutionary history is not known but can be carried out using simulated populations. Here we use simulated populations in which the true ancestry of all strains is known to provide a quantitative assessment of the performance of eBURST for populations evolving with differing rates of mutation and recombination.

## Results

### Quantitative assessment of eBURST

**Figure 1:**
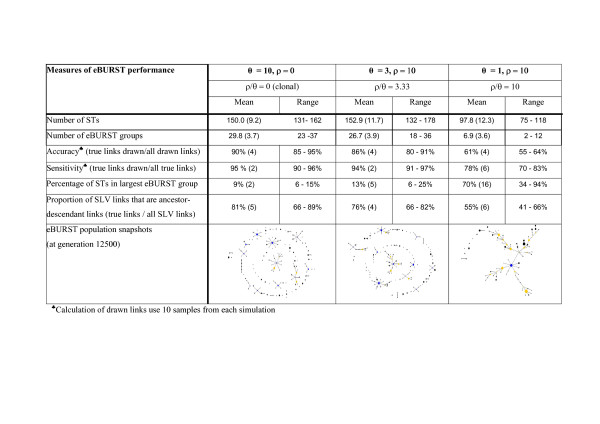
**Performance of eBURST for populations simulated with and without recombination**. The values are the averages and ranges from 20 samples of 500 isolates taken at 500 generation intervals from evolving populations of 1000 isolates with different values of the population mutation (θ) and recombination (ρ) rates.

**Figure 2:**
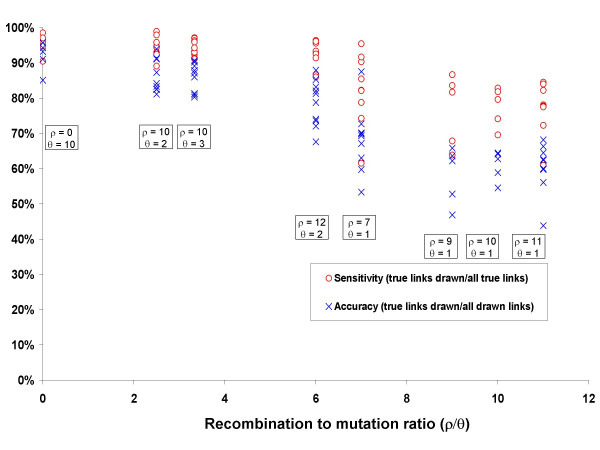
**Relationship between sensitivity and accuracy of eBURST and the recombination to mutation ratio**. For each parameter combination, 500 isolates were selected at random from the simulated population of 1000 isolates at 500 generation intervals after equilibrium had been reached. Accuracy and sensitivity are shown for individual samples from the simulations with different combinations of ρ and θ.

The inferred ancestor-descendant SLV links drawn by eBURST were compared to the known ancestor-descendant SLV links in populations simulated with different levels of recombination. The performance of eBURST was evaluated by measuring the proportion of the links that it draws that are correct (accuracy). As a method can be accurate but insensitive (e.g. if the links drawn are correct but many of the links that should be drawn are not drawn), the sensitivity of eBURST was also measured, as the proportion of correct SLV links that are drawn. These performance measures are summarised in Figure 1 for clonal populations and populations with moderate (ρ/θ = 3.3) and high (ρ/θ = 10) levels of recombination and are shown in Figure 2 for populations generated under clonality and for a range of values of the recombination to mutation (ρ/θ) ratio and

As expected, the best performance of eBURST was obtained under clonality, but moderate levels of recombination had little effect on the ability of eBURST to assign ancestor-descendant links correctly (Figure 1 and Figure 2). The accuracy was on average 86% with ρ/θ = 3.3, which was comparable to the 90% accuracy obtained in the clonal case. Increasing the ρ/θ ratio further resulted in a decline in eBURST performance and for ρ/θ = 10 the average accuracy dropped to 61%. The sensitivity of eBURST followed the same decreasing trend with increasing ρ/θ (Figure 2). The average sensitivity was 95% in the clonal case, 94% for ρ/θ = 3.3 and 78% for ρ/θ = 10(Figure 1).

### Comparison of eBURST to true ancestry and population structure with differing levels of recombination

The population structure (according to eBURST) was assessed for 20 samples of 500 isolates taken from populations of 1000 isolates simulated without recombination and with moderate and high ρ/θ ratios. Under clonality the population snapshots showed multiple radial eBURST groups, short chains and many individual STs and the largest group contained on average 9% of the STs in the population (Figure 1). For moderate ρ/θ the population snapshots were similar to the clonal case and the largest eBURST group included an average of 13% of the total STs. However, with a high ρ/θ ratio, the population had a noticeably different structure, typified by a single large group containing more than half the STs in the population (Figure 1; values for individual simulations using different values of ρ and θ are provided as supplementary material online, Additional file 2). These large groups typically have multiple linked radial groups and long chains of linked STs connecting one end of the eBURST group to the other. Consequently, STs at opposite ends of the large eBURST groups may have no alleles in common.

**Figure 3:**
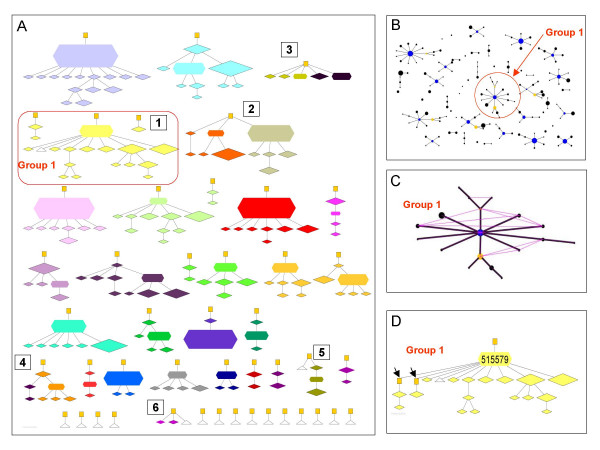
**Performance of eBURST for a population simulated in the absence of recombination**. All 1000 isolates from an equilibrium population, simulated with θ = 10 and ρ = 0, were displayed as A) the true ancestry groups that eBURST attempts to recover and B) eBURST groups. True ancestor-descendant relationships are shown in (A) by lines between the nodes and continuously connected groups of STs define the ancestry groups. The eBURST population snapshot (B) shows the clonal complexes and singletons. The largest eBURST group (Group 1) is labelled. C) Group 1 shows all of the additional SLVs (pink lines) overlaid on the eBURST diagram. D) Complete ancestry of the STs within eBURST Group 1 showing intermediate extinct STs (yellow squares). The isolates descending from the two extinct STs on the left (arrows) are in separate ancestry groups although they are in the same eBURST group (see text and supplementary online information). In A) node size is proportional to the frequency of an ST in the sample, and nodes are coloured by eBURST group. Nodes shaped as hexagons indicate the founders predicted by eBURST; diamonds are sampled STs; yellow squares are extinct ancestors of STs in the population; white triangles are singletons. In eBURST groups, the circles indicate STs and the area of each circle denotes the frequency of the ST. Blue circles denote the predicted founders of eBURST groups, yellow denotes a subgroup founder [11]. Black lines between STs show the inferred evolutionary relationships from the founder to the other STs in the eBURST group. Further description of Figure 3 is available as additional files.

**Figure 4:**
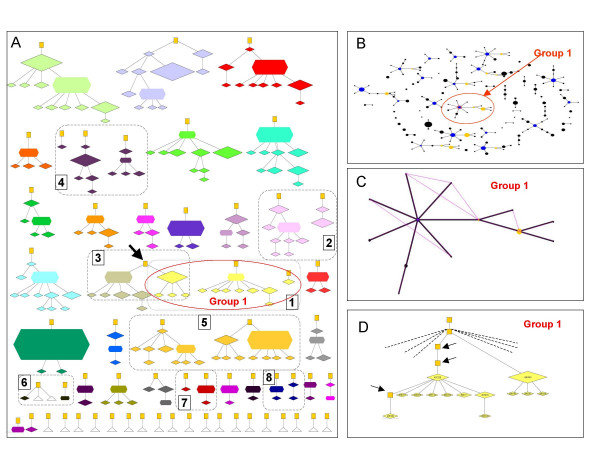
**Performance of eBURST for a population simulated with a moderate recombination to mutation ratio**. All 1000 isolates from an equilibrium population simulated with ρ = 10, θ = 3 were displayed as ancestry groups (A) and eBURST groups (B). C) The largest eBURST group (Group 1) is shown with all additional SLVs indicated. See Figure 3 for details. Further description of the eight discrepancies (numbered 1–8) between the ancestry groups and eBURST groups is available as additional files.

**Figure 5:**
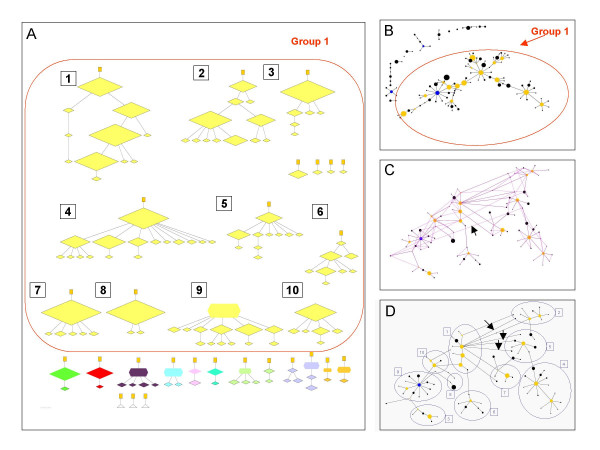
**Performance of eBURST for a population simulated with a high rate of recombination**. All 1000 isolates from an equilibrium population simulated with ρ = 10, θ = 1 were displayed as ancestry groups (A) and eBURST groups (B). The large eBURST group (Group 1) includes many unrelated ancestry groups, which are numbered. C) All of the additional SLV links are shown in pink for the largest eBURST group (Group 1). The arrow shows an example of a long-range SLV link. D) The groups of STs within eBURST Group 1 that correspond to the ancestry groups are shown, numbered as in (A). The eBURST group is the same at that in (B), except that subgroups and STs have been moved relative to each other to be able to show better the relationship with the ancestry groups. Arrows show examples of STs within a radial eBURST subgroup that should be in different ancestry groups. See Figure 3 for details. Further description of Figure 5 is available as additional files.

Typical eBURST snapshots of the complete population of 1000 isolates are illustrated, together with the groups expected from the true ancestry under clonality (Figure 3), moderate ρ/θ (Figure 4) and high ρ/θ (Figure 5). There are different types of error which result in discrepancies between the eBURST prediction and the true ancestry. Minor errors resulted in some isolates from the same ancestry group being split into two eBURST groups due to either a change at two loci in a single generation of the model, or missing (extinct or unsampled) intermediate STs. Major errors are defined as cases where STs that do not share a recent common ancestor are grouped into the same eBURST group (i.e. there are more than three generations in the ancestry to a common ancestor).

In the clonal example, eBURST groups corresponded closely to the true ancestry (the uniform colour of the nodes within almost all ancestry groups indicates the correspondence between the ancestry and eBURST groups), and there were only four minor (and no major) discrepancies out of 30 groups (Figure 3). For example, in Group 1 there are two ancestry groups that have been placed in a single eBURST group. Figure 3d shows how the isolates not joined to the main part of the ancestry group (but included in the eBURST group) are linked to the ancestry group via their extinct parents (indicated with black arrows). The changes of alleles between these isolates, their parents and common grandparent all occurred at the same locus. These changes at the same locus result in the isolates labelled with arrows being SLVs of their common grandparent, which joins them into the same eBURST group.

Even with a moderate ρ/θ ratio the majority of errors are minor and only three out of eight discrepancies between eBURST and the 35 ancestry groups were considered major, joining unrelated ancestry groups together (Figure 4). However, with the high ρ/θ ratio, 10 groups that do not share recent common ancestry were inappropriately linked into one large eBURST group (Figure 5a, b). Whilst the local radial subgroups in the large eBURST group corresponded in several cases to ancestry groups (Figure 5d), these subgroups should not be joined together. Further detailed description of Figures 3, 4, 5 and examples of the types of errors that eBURST may make are given in additional file 3.

### Relationship between eBURST performance and proportion of STs in the largest eBURST group

**Figure 6:**
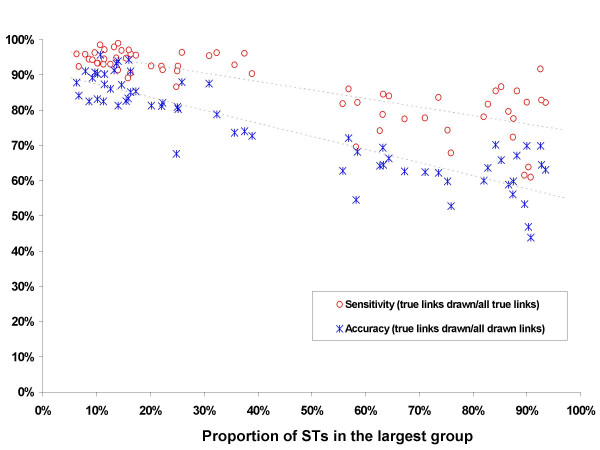
**Relationship between the performance of eBURST and the proportion of STs in the largest group**. Ten realisations of each simulation were generated with different combinations of ρ and θ. Random samples of 500 isolates were drawn from the population of 1000 isolates at 500 generation intervals after generation 5000.

There was considerable variation in eBURST performance between samples obtained with the same combination of theta (θ) and rho (ρ), which increased with increasing levels of recombination (Figure 1). However, there was a strong negative correlation between eBURST performance and the proportion of STs in the largest eBURST group that was insensitive to the variability between samples obtained with the same parameter values (Figure 6). When the proportion of STs in the largest eBURST group was between 5–25%, the proportion of the links drawn that have an ancestor-descendant relationship (the sensitivity) is over 90% (Figure 6). As the proportion of STs in the largest group increases, the accuracy and sensitvity of eBURST decrease approximately linearly.

### Evaluating the reliability of eBURST groups obtained using real MLST data

**Figure 7:**
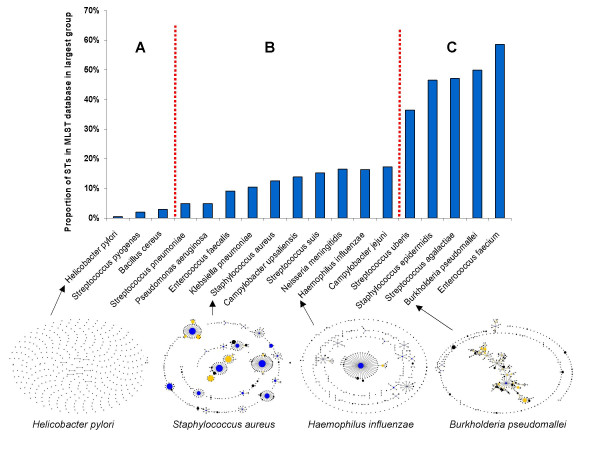
**Proportion of STs in the largest eBURST group for populations of species in the MLST databases**. All isolates in the MLST databases for a number of species were obtained from MLST [14] and pubMLST [15] websites and the proportion of STs in the largest eBURST group was calculated. eBURST population snapshots are shown for four selected species with differing proportions of STs in their largest eBURST group. In area A the population is so diverse that clonal complexes may not be apparent (see text), in area B eBURST performance should be good, whereas in area C the performance is likely to be poor due to high levels of recombination.

All isolates of each species represented at the two main MLST websites(MLST [14] and PubMLST [15]) were displayed as population snapshots and the proportion of STs in the largest eBURST group was calculated (Figure 7). Most species fall within the region where eBURST performs well (5–25% STs in the largest eBURST group) but five had between 37 and 59% of the STs in the largest eBURST group and were thus in the region where performance is likely to be poor.

The eBURST population snapshots are shown for selected species. In *Helicobacter pylori *eBURST provides no useful information on the patterns of descent among STs as there are very few pairs of SLVs and no larger clusters of linked STs in this very diverse, highly recombining species [16]. *Staphylococcus aureus *and *Haemophilus influenzae *provide examples of species for which eBURST performs very well and, respectively, are representative of species with low and moderate recombination to mutation ratios [17,18]. For *Burkholderia pseudomallei *there is one very large straggly eBURST group, indicating that groups of related isolates are likely to be spuriously linked into this large eBURST group [19]. This type of population snapshot was only observed in populations simulated with high recombination to mutation ratios and, as predicted, recent evidence suggests high rates of recombination, but low allelic diversity in *B. pseudomallei *[20,21]. A single large straggly eBURST group was also found in other species, for example *Enterococcus faecium *and *Streptococcus uberis *(data not shown), and recombination is also predicted to be frequent relative to mutation in these populations [22,23]. The overall topologies of the population snapshots from simulations are remarkably consistent with those obtained for species with similar empirically estimated recombination to mutation ratios (Figure 1).

## Discussion

The eBURST program is widely used but its ability to correctly identify clonal complexes, and to discern patterns of descent within clonal complexes, for populations with different levels of recombination, has not been assessed. There are a number of ways in which eBURST could be unreliable, the most serious of which is linking together groups of STs that are not closely related. As expected, for strictly clonal populations, eBURST groups corresponded very closely to the ancestry groups and the accuracy and sensitivity of eBURST was on average ≥ 90%.

With occasional exceptions, where groups of strains that did not share recent ancestry were incorrectly linked into a single eBURST group, the performance of eBURST remained good for populations with moderate levels of recombination, but spurious linking together of clonal complexes into one large eBURST group was observed in populations with high recombination to mutation ratios. As well as correctly identifying groups of related STs, the ability of eBURST to identify correct ancestor-descendant links within these groups was high in clonal populations and remained high with low or moderate levels of recombination, decreasing approximately linearly with increasing ρ/θ ratios. However, at ρ/θ = 10 the accuracy of eBURST dropped to about 60%.

The spurious linking together of clonal complexes with high rates of recombination resulted in populations that were dominated by a single large straggly eBURST group, which typically include a number of radial subgroups linked through chains of SLVs. The radial subgroups corresponded reasonably well with the ancestry groups (Figure 5d), suggesting these largely reflect simple patterns of descent from their subfounder STs [11] and that it is the interlinking of the radial subgroups that is incorrect. In essence, these radial subgroups approximate the real clonal complexes, which are joined together inappropriately. However, examination of Figure 5d also shows multiple examples of individual STs within radial subgroups that are wrongly placed.

Even in strictly clonal populations not all SLVs represent true ancestor-descendant links (Figure 1) since two SLVs of an ancestor can arise by different changes at the same locus, resulting in STs that are SLVs of each other, but which do not have an ancestor-descendant relationship (Figure 3c). It is the difficulty in deciding which of these alternative SLV links represent the real pattern of descent that prevents eBURST from being 100% accurate even in the absence of any recombination. As recombination becomes more frequent relative to mutation, an increasing proportion of SLVs in the population do not represent ancestor-descendant links (Figure 1). Displaying these additional undrawn SLV links in a population with a high ρ/θ shows there typically are SLV links that extend across the single large eBURST group found in such populations (Figure 5c). These long-range SLV links occur in these populations as STs may share many alleles, not due to common ancestry, but to very frequent recombination (13). Incorrect linking of subgroups, and of individual SLVs, will occur in a large eBURST group when long-range SLV links are observed since, besides the SLV links that are drawn by eBURST, there will in some cases be alternative undrawn links between SLVs far apart in the eBURST group. Choosing one SLV link as the predicted true ancestry, rather than the alternative link, will lead to major differences in the linking of subgroups or individual STs. In eBURST the algorithm is to link SLVs to the ST with the largest number of SLVs first, then go to the ST with the next highest number of SLVs that have not previously been linked, and link these and so on. If two STs have the same number of SLVs, the one with the largest number of DLVs is selected [12]. This difficulty in choosing between the large numbers of alternative SLV links in populations with high rates of recombination makes accurate reconstruction of recent ancestry problematic using eBURST, or any other method that uses allelic data.

Methods that use the sequences rather than alleles face the challenge of identifying the true source of each piece of variable sequence as mutant or recombinant. In the case of one recent method, ClonalFrame [24], this is sidestepped by assuming that recombination only involves importation of alleles (sequences) from outside of the dataset. However, the problem with inferring ancestry using MLST data in populations with high recombination to mutation ratios is the frequent importation ofexisting sequences from unrelated strains present in the dataset, which generates strains that are similar due to recombination rather than descent. Such methods are hence unlikely to be helpful in this sort of situation.

It should be stressed that the existence of STs that share several alleles due to recombination rather than common ancestry, leading to straggly eBURST groups, occurs when high levels of recombination occur in populations with relatively small numbers of alleles. If there are large numbers of alleles in the population (e.g. due to frequent generation of new alleles as a consequence of a high mutation rate), high rates of recombination will not generate unrelated STs that share several alleles, but a very diverse population in which there are no clonal complexes, since few isolates in the population will differ at only a single locus. *H. pylori *provides a good example of this type of highly diverse recombining population (Figure 7), which is consistent with the high rate of mutation that generates large numbers of alleles and a high rate of recombination that shuffles these alleles (13, 20). As expected, populations simulated with high rates of both mutation and recombination generated populations that produce very similar population snapshots to that shown for *H. pylori *in Figure 7 (data not shown).

How can the reliability of eBURST be judged when applied to real MLST data from a bacterial population? The proportion of STs in the largest group was identified as a robust indicator of eBURST performance. This measure insensitive to variation in the performance of eBURST observed between different samples obtained from the same simulation. It is also very straightforward to calculate from the analysis window within eBURST and does not require any prior knowledge of the extent of recombination in the population. If the largest eBURST group contains more than 25% of the STs in the population, eBURST performance is likely to be suboptimal in terms of predicting ancestor-descendant links and, more importantly, may join unrelated groups of STs into the same eBURST group.

The presence of a single large straggly eBURST group is also a useful indicator that clonal complexes have been inappropriately linked and that there may be a high recombination to mutation ratio (ρ/θ) within the population. Further suggestive evidence for high ρ/θ can be obtained when the largest eBURST group has many long-range SLV links and chains of STs connecting radial subgroups. The presence of a single large eBURST group in real populations of the five species in area C of Figure 7 immediately suggests that eBURST will be unreliable and that the ratio of recombination to mutation is likely to be high in these species.

The overall topologies of the eBURST population snapshots for different bacterial species are consistent with those obtained in the simulations and proportion of STs in the largest group appears to be a reasonable proxy for the recombination to mutation ratio, where this has been estimated. Ideally, population snapshots should be based on a large unbiased sample of the population. The population snapshots in Figure 7 are taken from the entire MLST databases, which contain variable numbers of isolates and in many cases have biased sampling (e.g. oversampling of isolates that are antibiotic-resistant or from serious disease). The major consequence of oversampling is to identify large numbers of isolates of the oversampled STs and this has no effect on the structure of eBURST groups. The number of STs may also increase to some extent due to oversampling, as minor variants of oversampled STs are more likely to be sampled, but these will be SLVs of the oversampled STs. However, more SLVs of a few clones due to oversampling is not going to make a straggly eBURST group a radial group, or vice versa.

MLST databases (or population samples) should be relatively large to get an indication of the reliability of eBURST, or the presence of a dominant straggly group, rather than radial groups. Analysis of subsets of isolates from the entire MLST databases for *B. pseudomallei *and *E. faecium *showed that a dominant straggly eBURST group was observed in substantially smaller samples than the entire MLST databases. Thus, the population snapshots of the first 200 isolates, or the second 200 isolates, from the *B. pseudomallei *and *E. faecium *MLST databases gave population snapshots that were similar to those in Figure 7, being dominated by a single large straggly eBURST group. Similarly, radial eBURST groups were present in samples of the first 200 and the second 200 isolates taken from the *S. aureus *and *H. influenzae *MLST databases (data not shown).

The ρ/θ ratio in the neutral, infinite alleles, model is not directly equivalent to the recombination/mutation (r/m) ratio obtained from MLST data using the method of Feil et al [8], since θ is a parameter that incorporates all processes generating new alleles (see Methods), and ρ/θ values are therefore lower than r/m values. The good reliability of eBURST up to values of about ρ/θ = 4 implies it will be reliable for species with r/m values considerably higher than 4:1, and this is consistent with the predicted good performance of eBURST for *Neisseria meningitidis *and *Streptococcus pneumoniae *(Figure 5), which have r/m values of about 5–9 [9].

This initial analysis considered populations evolving under neutrality, sampled randomly at equilibrium. In experimental data there will be deviations from these simplifying assumptions, including selection, sampling bias, growing or declining populations, which may result in distinctive features in the population snapshot that have not been uncovered in this analysis. The approach used here can easily be extended to consider the reliability of eBURST or similar methods under more complex evolutionary scenarios. However, for populations that are not highly biased, the > 25% guideline is a useful indicator of poor eBURST performance and none of the bacteria surveyed in the MLST database were inconsistent with this assertion.

Other approaches have been developed for the analysis of MLST data and a method based on minimum-spanning trees is incorporated into the Bionumerics™ package [25]. This method incorporates the BURST algorithm for closely-related STs, but links groups of related STs (clonal complexes) to each other through hypothetical missing intermediate STs, to produce a representation of the whole population in which all STs are linked. The robustness of these linkages between clonal complexes has not been evaluated, but given that only local structure remains reliable in eBURST under high rates of recombination, our experience would suggest that many of these inferred links between clonal complexes produced by postulating missing intermediates will be spurious if recombination rates are moderate or high. Links between clonal complexes through postulated intermediates may also be spurious under strict clonality, since in such populations different lineages diverge without bound, and STs in different complexes may share few or no alleles. In the latter situation, analysis of the sequences rather than the allelic profiles would be expected to give a more reliable indicator of the relationships between clonal complexes, using standard phylogenetic methods. An analysis of the robustness of minimum-spanning trees using populations simulated with different levels of recombination would be worthwhile.

## Conclusion

eBURST provides a robust picture of bacterial populations over a wide range of ρ/θ parameters, only becoming seriously unreliable with high rates of recombination, and by focusing on identifying and exploring descent within clonal complexes is a conservative and cautious approach. We provide three simple checks which may indicate high ρ/θ and hence poor reliability (Table 1). As discussed previously, eBURST groups should be considered to be hypotheses about ancestry and patterns of descent among similar STs and additional data should be used to explore the validity of the inferred relationships [11].

## Methods

### Simulating bacterial populations

Bacterial populations were simulated using the neutral, infinite alleles, model of Fraser et al [26]. The model assumes non-overlapping generations, with subsequent generations selected by sampling with replacement from the current one, i.e. it is a stochastic process where the probability of a sequence type (ST) occurring in the next generation is proportional to its frequency in the current generation. At each generation, alleles can change at defined rates by mutation or recombination. Under the infinite alleles assumption, mutation always generates a new allele. The mutation parameter θ includes point mutation and also any other process that generates new alleles in real populations (e.g. mosaic alleles formed by recombination or importation by recombination of alleles from outside the population). Recombination introduces an existing allele randomly selected from the isolates present in the previous generation, which may generate a novel allelic profile (new ST), whereas mutation always generates a new ST. Mutation or recombination occur independently at each locus. Each event is rare, so typically a new descendant ST shares alleles at all but one locus with its immediate ancestor. When a new ST is produced, by mutation or recombination, it is given a new ST number and the parental ST is recorded. For new STs generated by recombination, the ST that donated the allele, and the locus involved, is also recorded. The evolution of the simulated population over time is shown diagrammatically in additional file 1.

Simulations were performed with strains (STs) defined as in MLST, by the alleles at seven loci, and a range of values for the population mutation rate (θ) and the population recombination rate (ρ). These parameters are functions of the population size (N) and the mutation rate (m) and recombination rate (r), as follows [26]:

θ = 2mN

ρ = 2rN

Under the neutral model the population structure reaches a dynamic equilibrium, in which the rate of generation of new STs is balanced by the stochastic extinction of STs. The diversity of simulated populations is determined by the mutation and recombination rate. The populations (N = 1000) were allowed to evolve, with a range of values of θ and ρ. The stability of the index of sequence type diversity (or homozygosity) (defined as

H=(1−∑i=1sxi2)(NN−1)
 MathType@MTEF@5@5@+=feaafiart1ev1aaatCvAUfKttLearuWrP9MDH5MBPbIqV92AaeXatLxBI9gBaebbnrfifHhDYfgasaacH8akY=wiFfYdH8Gipec8Eeeu0xXdbba9frFj0=OqFfea0dXdd9vqai=hGuQ8kuc9pgc9s8qqaq=dirpe0xb9q8qiLsFr0=vr0=vr0dc8meaabaqaciaacaGaaeqabaqabeGadaaakeaacqWGibascqGH9aqpdaqadaqaaiabigdaXiabgkHiTmaaqahabaGaemiEaG3aa0baaSqaaiabdMgaPbqaaiabikdaYaaaaeaacqWGPbqAcqGH9aqpcqaIXaqmaeaacqWGZbWCa0GaeyyeIuoaaOGaayjkaiaawMcaamaabmaabaWaaSaaaeaacqWGobGtaeaacqWGobGtcqGHsislcqaIXaqmaaaacaGLOaGaayzkaaaaaa@42E5@

where *x*_*i *_is the frequency of the *i*^th ^ST, *s *is the number of STs and *N *is the total number of isolates in the sample), and other measures (e.g. the number of STs), were assessed each generation to ensure that an equilibrium population structure had been achieved prior to sampling. After reaching equilibrium, random samples of 500 isolates were taken every 500 generations of the simulation, to obtain independent samples.

An application written in JAVA was used to run and take samples from the simulation model (written in C++), to analyse the samples (using the JAVA application eBURST v3,[12]), and to compare the results with the known ancestry. For each isolate sampled from the simulation, its identifier (sequence type, ST), the ST of its immediate ancestor, its age in model generations, its allelic profile and its complete ancestry since the most recent universal common ancestor of the population were known.

In the fully clonal case (no recombination, ρ = 0), simulated populations of 1000 isolates were run to equilibrium with θ = 10, (equivalent to m = 0.005). The effect of introducing allelic change by recombination on the reliability of eBURST was explored by preliminary analyses to identify regions of interest in parameter space. As the recombination to mutation ratio increased above 10:1, the performance of eBURST declined, and a ratio of 14:1 was selected as the upper limit for simulations (additional file 2).

For each sample of the simulated population, the number of STs, the eBURST groups, their predicted founding STs, and patterns of descent were obtained. A correctly inferred eBURST SLV link joins two STs that, from examination of the real ancestry, have an ancestor-descendant relationship. The direction of the relationship is not considered. For selected parameter combinations, the performance of eBURST was also assessed quantitatively for 10 independent samples. Population snapshots were displayed using eBURST v3 with the default settings. Additional SLVs, that were not predicted by eBURST to represent ancestor-descendant relationships (and thus were not drawn), were displayed on eBURST diagrams using features available within eBURST v3.

Performance of eBURST was evaluated by measuring its accuracy and sensitivity in identifying the SLVs that have true ancestor-descendant relationships. Accuracy was defined as the proportion of links drawn between SLVs in an eBURST population snapshot that have an ancestor-descendant relationship:

True SLV links drawnTotal SLV links drawn (i.e. correct and incorrect links)
 MathType@MTEF@5@5@+=feaafiart1ev1aaatCvAUfKttLearuWrP9MDH5MBPbIqV92AaeXatLxBI9gBaebbnrfifHhDYfgasaacH8akY=wiFfYdH8Gipec8Eeeu0xXdbba9frFj0=OqFfea0dXdd9vqai=hGuQ8kuc9pgc9s8qqaq=dirpe0xb9q8qiLsFr0=vr0=vr0dc8meaabaqaciaacaGaaeqabaqabeGadaaakeaadaWcaaqaaiabbsfaujabbkhaYjabbwha1jabbwgaLjabbccaGiabbofatjabbYeamjabbAfawjabbccaGiabbYgaSjabbMgaPjabb6gaUjabbUgaRjabbohaZjabbccaGiabbsgaKjabbkhaYjabbggaHjabbEha3jabb6gaUbqaaiabbsfaujabb+gaVjabbsha0jabbggaHjabbYgaSjabbccaGiabbofatjabbYeamjabbAfawjabbccaGiabbYgaSjabbMgaPjabb6gaUjabbUgaRjabbohaZjabbccaGiabbsgaKjabbkhaYjabbggaHjabbEha3jabb6gaUjabbccaGiabcIcaOiabbMgaPjabb6caUiabbwgaLjabb6caUiabbccaGiabbogaJjabb+gaVjabbkhaYjabbkhaYjabbwgaLjabbogaJjabbsha0jabbccaGiabbggaHjabb6gaUjabbsgaKjabbccaGiabbMgaPjabb6gaUjabbogaJjabb+gaVjabbkhaYjabbkhaYjabbwgaLjabbogaJjabbsha0jabbccaGiabbYgaSjabbMgaPjabb6gaUjabbUgaRjabbohaZjabcMcaPaaaaaa@8AB6@

Sensitivity was defined as the proportion of links drawn which have an ancestor-descendant relationship:

True SLV links drawnTotal correct SLV links (i.e. drawn and not drawn)
 MathType@MTEF@5@5@+=feaafiart1ev1aaatCvAUfKttLearuWrP9MDH5MBPbIqV92AaeXatLxBI9gBaebbnrfifHhDYfgasaacH8akY=wiFfYdH8Gipec8Eeeu0xXdbba9frFj0=OqFfea0dXdd9vqai=hGuQ8kuc9pgc9s8qqaq=dirpe0xb9q8qiLsFr0=vr0=vr0dc8meaabaqaciaacaGaaeqabaqabeGadaaakeaadaWcaaqaaiabbsfaujabbkhaYjabbwha1jabbwgaLjabbccaGiabbofatjabbYeamjabbAfawjabbccaGiabbYgaSjabbMgaPjabb6gaUjabbUgaRjabbohaZjabbccaGiabbsgaKjabbkhaYjabbggaHjabbEha3jabb6gaUbqaaiabbsfaujabb+gaVjabbsha0jabbggaHjabbYgaSjabbccaGiabbogaJjabb+gaVjabbkhaYjabbkhaYjabbwgaLjabbogaJjabbsha0jabbccaGiabbofatjabbYeamjabbAfawjabbccaGiabbYgaSjabbMgaPjabb6gaUjabbUgaRjabbohaZjabbccaGiabcIcaOiabbMgaPjabb6caUiabbwgaLjabb6caUiabbccaGiabbsgaKjabbkhaYjabbggaHjabbEha3jabb6gaUjabbccaGiabbggaHjabb6gaUjabbsgaKjabbccaGiabb6gaUjabb+gaVjabbsha0jabbccaGiabbsgaKjabbkhaYjabbggaHjabbEha3jabb6gaUjabcMcaPaaaaaa@8292@

To assess the integrity and inclusiveness of eBURST groups, we used the known ancestry to define an ideal eBURST group as a group of STs in the sample continuously connected by ancestor-descendant links. This provides the groupings, founders and patterns of local descent that eBURST should recover, and can be visualised as network graphs, with node colour representing the eBURST group to which each ST is assigned (XML available on request from Tom Connor). The ability of eBURST to recapture the true pattern of recent descent is therefore an indicator of overall performance. Closer comparison between the eBURST groups and those expected from the known ancestry (ancestry groups) can also illustrate the types of errors made by eBURST.

The eBURST population snapshots obtained from simulated populations generated with differing ratios of recombination to mutation were compared to those obtained for real bacterial populations by analysing all isolates within each of the online MLST species databases (MLST [14] and PubMLST [15]), using the links to these databases provided through the eBURST v3 website [27].

## Authors' contributions

KT planned, designed and undertook all the analyses, wrote the JAVA code to implement analysis and wrote the first draft of the paper, CF wrote the simulation model, TC wrote the XML code for displaying the true ancestry groups, BS conceived and coordinated the study, and co-wrote the submitted manuscript, WH, CF and BS participated in the study design and interpretation of results and all authors critically appraised for intellectual content and approved the final draft.

## Supplementary Material

Additional File 2 Two measures of the overall population structure. The proportion of all SLVs that have an ancestor-descendant relationship (A), and the proportion of STs in the largest eBURST group (B), were calculated for populations simulated with different recombination and mutation parameters. For each parameter combination, twenty samples (500 isolates) were taken at intervals from the simulations after burn-in. The red crosses in the two top graphs are the values for the clonal populations. Click here for file

Additional file 3 Further explanation of the comparison between eBURST groups and the true ancestry, to illustrate the types of errors made by eBURST, illustrated in Figures 3, 4, 5. Click here for file

Additional file 1 Evolution of a simulated population of bacteria. Only five isolates are shown, the seven digits corresponding to the allele numbers at the seven MLST loci. At generation *t+1 *isolates are selected at random from generation *t*, with mutation having occurred between generations in one isolate, resulting in a new allele and a new ST (allelic profile) in generation *t+1*. In generation *t+2 *a new ST has arisen by mutation, and recombination has replaced allele 4 in an isolate from generation t+1 with allele 1 from another of the isolates, to produce another new ST in generation *t+2*. After many generations the population reaches a dynamic equilibrium (*t+n*) in which the STs present still change over time but the overall population structure remains the same. Click here for file

## Figures and Tables

**Table 1:** 

Simple tests for populations in which eBURST is likely to be unreliable
1) Display population snapshot – is there a single large straggly group?
2) Display undrawn SLV links – are there long range SLV links across this group?
3) Calculate proportion of STs in the largest group – is it greater than 25%?
If the answer to all three questions is yes, then there is probably a high rate of recombination compared to mutation and the performance of eBURST will be poor.
