# Supplementary figures and images for: Assessing the reliability of eBURST using simulated populations with known ancestry
Source: BMC Microbiol. 2007 Apr 12;7:30. doi: 10.1186/1471-2180-7-30 (PMC1865383; doi:10.1186/1471-2180-7-30)

## Slide 1
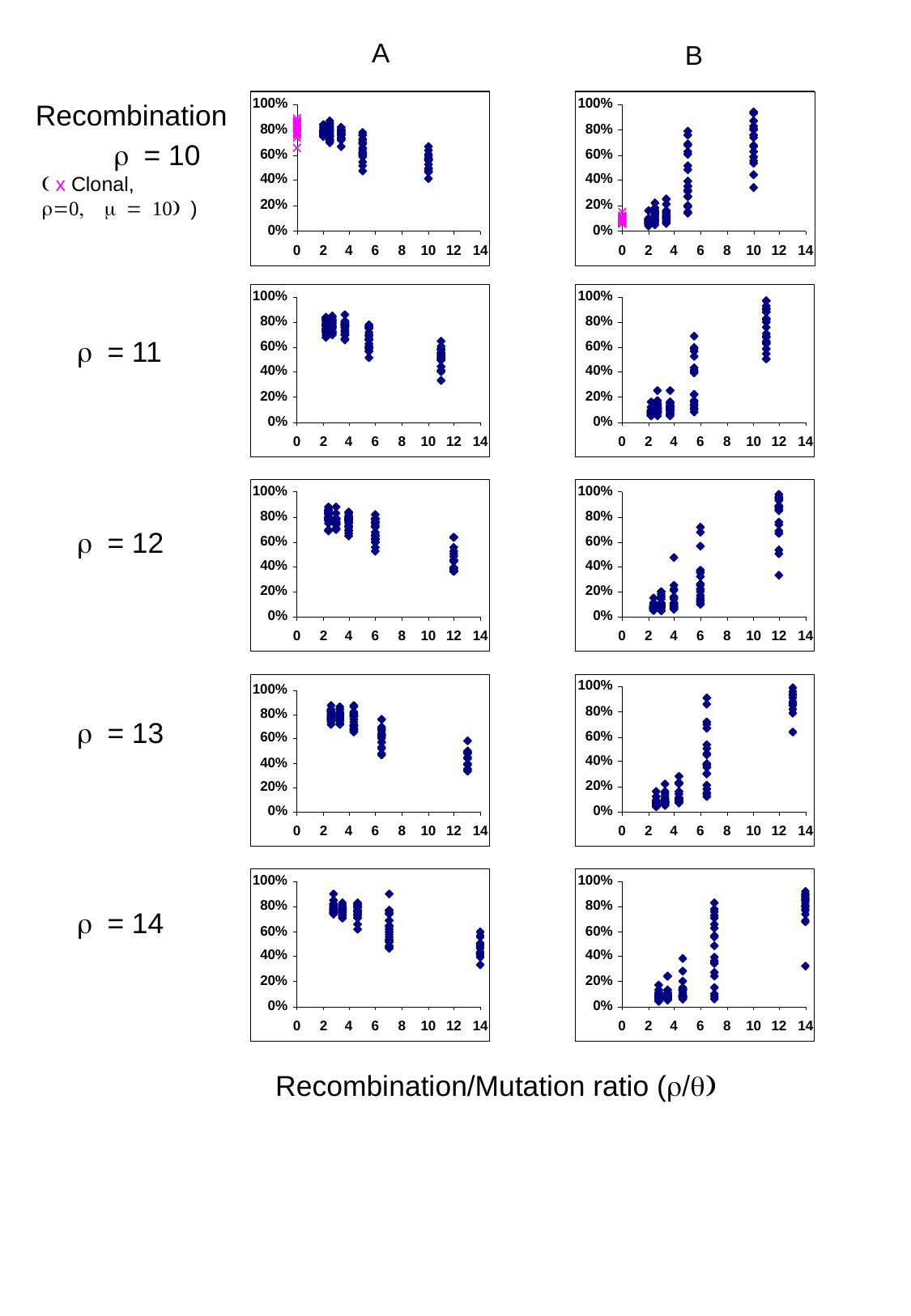

A
B
Recombination
  = 10
 x Clonal,   =  )
 = 11
 = 12
 = 13
 = 14
Recombination/Mutation ratio (

Supplement: Additional File 2 — Two measures of the overall population structure. The proportion of all SLVs that have an ancestor-descendant relationship (A), and the proportion of STs in the largest eBURST group (B), were calculated for populations simulated with different recombination and mutation parameters. For each parameter combination, twenty samples (500 isolates) were taken at intervals from the simulations after burn-in. The red crosses in the two top graphs are the values for the clonal populations. [file 1471-2180-7-30-S2.ppt]

## Slide 1
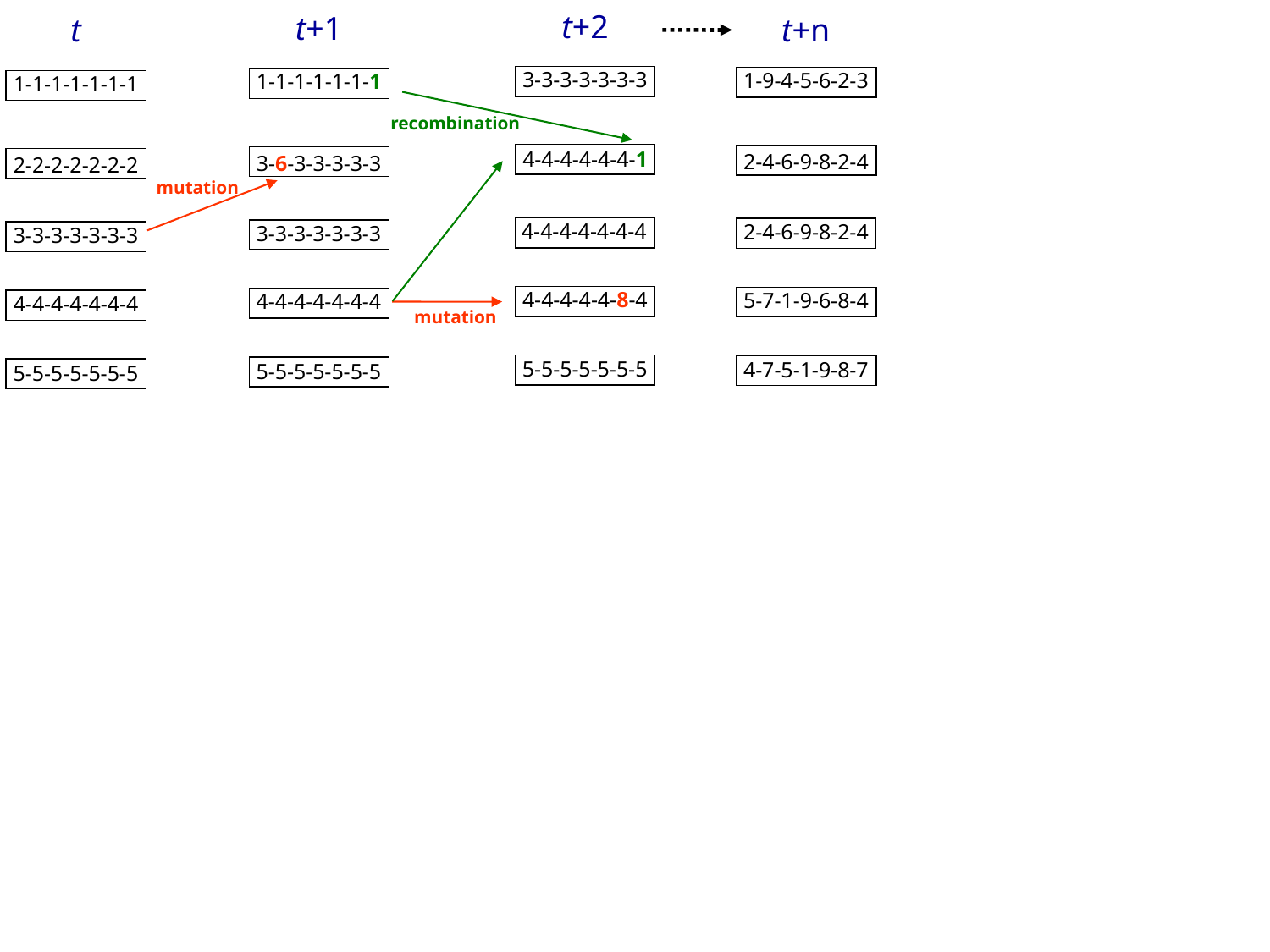

t+2
3-3-3-3-3-3-3
4-4-4-4-4-4-1
4-4-4-4-4-4-4
4-4-4-4-4-8-4
5-5-5-5-5-5-5
t+1
1-1-1-1-1-1-1
3-6-3-3-3-3-3
3-3-3-3-3-3-3
4-4-4-4-4-4-4
5-5-5-5-5-5-5
t
1-1-1-1-1-1-1
2-2-2-2-2-2-2
3-3-3-3-3-3-3
4-4-4-4-4-4-4
5-5-5-5-5-5-5
t+n
1-9-4-5-6-2-3
2-4-6-9-8-2-4
2-4-6-9-8-2-4
5-7-1-9-6-8-4
4-7-5-1-9-8-7
recombination
mutation
mutation

Supplement: Additional file 1 — Evolution of a simulated population of bacteria. Only five isolates are shown, the seven digits corresponding to the allele numbers at the seven MLST loci. At generation t+1 isolates are selected at random from generation t, with mutation having occurred between generations in one isolate, resulting in a new allele and a new ST (allelic profile) in generation t+1. In generation t+2 a new ST has arisen by mutation, and recombination has replaced allele 4 in an isolate from generation t+1 with allele 1 from another of the isolates, to produce another new ST in generation t+2. After many generations the population reaches a dynamic equilibrium (t+n) in which the STs present still change over time but the overall population structure remains the same. [file 1471-2180-7-30-S1.ppt]
