# Supplementary material for: Assessing the reliability of eBURST using simulated populations with known ancestry
Source: BMC Microbiol. 2007 Apr 12;7:30. doi: 10.1186/1471-2180-7-30 (PMC1865383; doi:10.1186/1471-2180-7-30)
Supplement: Additional file 3 — Further explanation of the comparison between eBURST groups and the true ancestry, to illustrate the types of errors made by eBURST, illustrated in Figures 3, 4, 5. [file 1471-2180-7-30-S3.doc]

**Further details of comparison of eBURST groups with the ancestry groups, to accompany Figs 2-4.** The ability of eBURST to correctly identify groups of strains that have diversified from a recent common ancestor was evaluated. The eBURST groups in simulated populations of 1000 isolates were displayed as a population snapshot and, by using the known ancestry, were compared with the groups that should have been obtained. For the strictly clonal population (no recombination), the eBURST groups in the population snapshot demonstrate the classic radial pattern indicative of clonal complexes arising from the diversification of a founding ST (Figure 2b) [11]. True ancestral groups are defined as a group continuously connected by direct ancestor-descendant links. Each node in the ancestry group represents a single ST, the area representing its frequency in the population. The colours of the nodes in the true group (Figure 2a) represent the different groups to which the STs were assigned by eBURST; the high level of consistency of the colours of STs within each group in the diagram shows that the eBURST groups correspond well to the ancestry groups.

There were 4 minor discrepancies in the clonal population; three due to groups being split and 1 due to groups being joined together. Inspection of the complete ancestry showed that, even in the strictly clonal population, there were a few cases (3/30) where isolates in a single ancestry group were split into two eBURST groups (visualised in Figure 2a as a group containing STs of more than one colour). This occurred either because the common ancestor of the two eBURST groups that would link them together was extinct (e.g Figure 2a Groups 2 and 3), or because an ST had arisen from its immediate ancestor in a single generation of the model by two mutations in different loci (i.e. the descendant was a DLV of its immediate ancestor) (true ancestry Group 4). Extinction of the common ancestor in the ancestry groups 5 and 6 also excluded a single ST from the corresponding eBURST group and in Group 1 a single ST was excluded from the eBURST group as it arose as a DLV of its ancestor (shown as white triangles in the ancestry groups).

There was one example of an eBURST group joining ancestry groups together (Group 1, Figure 2a, 2b). Figure 2d shows how the isolates not joined to the main part of the ancestry group (but included in the eBURST group) can be linked via their extinct parents (indicated with black arrows). The true ancestry (Figure 2d) revealed that STs in this eBURST group do all have a recent common ancestry, but two linking STs were extinct. However, eBURST does place them in a single group as both the extinct STs and their descendant STs differed from the common ancestor (Figure 2d; strain 515579) at the same locus; thus the descendant STs are linked by eBURST to strain 515579 as they are SLVs of the latter strain as well as the two extinct intermediate STs.

For moderate levels of recombination (ρ/θ = 3.3) the eBURST groups usually corresponded to the true ancestry groups, indicating that eBURST generally identified groups of STs related by recent descent (Figure 3a, b). From 35 ancestry groups, there were eight cases where there were discrepancies with eBURST groups (Figure 3a). Examination of the complete ancestry showed that three of these cases were trivial (boxes 6, 7, 8 in Figure 3a), as the ancestry groups assigned to the same eBURST group were indeed closely related, but were not joined due to an extinct intermediate strain.. In one instance (box 3), an ancestry group was divided into two eBURST groups, due to an extinct common parent (arrow). eBURST Group 1 (box 1) consists of 3 ancestry groups which can be linked via a small number of intermediates (≤ 3 between ancestry groups), and therefore can be considered closely related (Figure 3d). In the remaining three cases (boxes 2, 4 5) the ancestry groups should not have been linked into a single eBURST group as they have no recent common ancestor (i.e. there are many more than 3 intermediates). Recombination has resulted in an SLV link between STs in the ancestry groups, which has wrongly joined them into a single eBURST group.

For the population simulated with a high level of recombination (ρ/θ  = 10), a high proportion of STs (47%) were within a single large eBURST group (Group 1; Figure 4b), which included ten ancestry groups (and four single STs) that should not be placed in the same eBURST group as they do not share recent ancestry (Figure 4a). Figure 4d shows the large eBURST group in more detail, with the STs assigned to the ten ancestry groups indicated. With some exceptions, the individual subgroups within the large eBURST group correspond fairly well with the groups defined by true ancestry, but these subgroups are incorrectly joined into a single large eBURST group.
